# A Rare Pediatric Neurological Case: Dyke-Davidoff-Masson Syndrome Unprecedented at 10 Months

**DOI:** 10.7759/cureus.53168

**Published:** 2024-01-29

**Authors:** Rahul Khandelwal, Jayant D Vagha, Revat J Meshram, Ankita Patel, Sri Sita Naga Sai Priya K

**Affiliations:** 1 Pediatrics, Jawaharlal Nehru Medical College, Datta Meghe Institute of Higher Education and Research, Wardha, IND

**Keywords:** seizures, ventricular enlargement, cerebral hemiatrophy, pediatric neurology, dyke-davidoff-masson syndrome

## Abstract

This case report presents a rare occurrence of Dyke-Davidoff-Masson Syndrome (DDMS) in a 10-month-old male child, highlighting the atypical presentation of this neurological disorder in early infancy. The child initially presented with irritability, loss of appetite, and right-sided weakness following episodes of fever. A comprehensive medical history revealed the sudden onset of generalized tonic-clonic seizures, prompting further investigation. Diagnostic imaging, including CT and MRI, confirmed features consistent with DDMS, including cerebral hemiatrophy, ventricular enlargement, and calvarial thickening. Notably, the child's seizures were successfully managed with antiepileptic medication, leading to stabilized vital signs. This case emphasizes the importance of considering rare neurological disorders in pediatric patients with unusual presentations and underscores the challenges in diagnosing and managing DDMS in infancy. Further research is warranted to elucidate the underlying mechanisms, contributing factors, and optimal management strategies for DDMS in this age group.

## Introduction

Dyke-Davidoff-Masson Syndrome (DDMS), first described by Dyke, Davidoff, and Masson in 1933, is an uncommon neurodevelopmental disorder characterized by cerebral hemiatrophy, ventricular enlargement, and calvarial thickening, often presenting with neurological complications such as seizures and hemiparesis [1]. Since 1933, when it was described for the first time, almost 100 cases have been reported [1]. Typically reported in older children and adolescents, DDMS is rarely observed in infants, making its presentation in a 10-month-old child particularly noteworthy [2]. The pathogenesis of DDMS is diverse, with vascular insult, infection, and trauma implicated as potential etiological factors [3]. Cerebral hemiatrophy, a key feature of DDMS, is thought to result from insults to the developing brain during early childhood, disrupting normal neuronal and vascular development [4]. The clinical manifestations of DDMS can vary widely, making accurate diagnosis challenging, especially in pediatric populations.

In this case, the child's clinical course began with episodes of fever at seven months, managed with IV antipyretics and antibiotics. Subsequently, the sudden onset of generalized tonic-clonic seizures and neurological deficits prompted further investigation, leading to the diagnosis of DDMS. The early onset of DDMS in infancy, as seen in this case, underscores the importance of considering rare neurological disorders in the differential diagnosis of pediatric patients presenting with atypical symptoms. Imaging plays a pivotal role in confirming the diagnosis of DDMS. CT and MRI are essential modalities for visualizing cerebral hemiatrophy, ventricular enlargement, and associated structural abnormalities [5]. These imaging findings aid in distinguishing DDMS from other conditions presenting with similar neurological manifestations. The management of DDMS involves a multidisciplinary approach, addressing the underlying cause and symptom control. Antiepileptic medications are commonly prescribed to manage seizures, while physiotherapy and rehabilitation may be necessary to address neurological deficits [6]. However, given the rarity of DDMS in infancy, more evidence is needed for optimal management strategies in this age group.

## Case presentation

**Figure 1:**
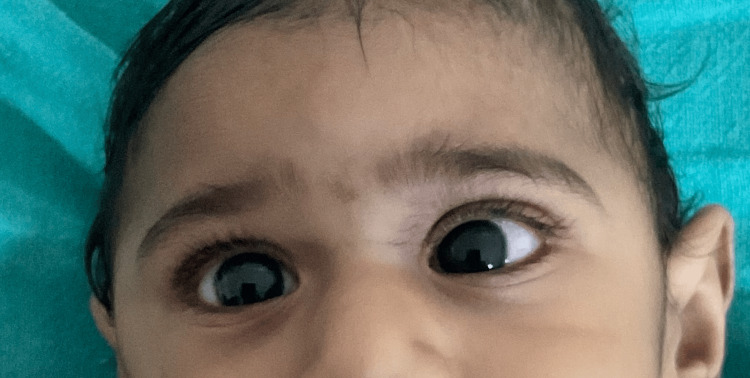
Facial asymmetry

We present a case involving a 10-month-old male child who was brought to the outpatient department of a tertiary care hospital. The child's primary complaints included frequent crying, irritability, loss of appetite persisting for 15 days, and weakness on the right side over the past month. Upon taking the child's medical history, the mother reported that the child had achieved developmental milestones and was normal until seven months old. Subsequently, the child had two episodes of fever lasting two days, which were managed with IV antipyretics and antibiotics at a local hospital. On the day of discharge from the local hospital, the child suddenly experienced multiple episodes of generalized tonic-clonic seizures, lasting two to three minutes each. These seizures involved stiffening of the right upper extremity, leading to the initiation of antiepileptic medication by the attending physician. During the physical examination, weakness, reduced sensation, and facial asymmetry were observed. As a result, the physician recommended admitting the child for further medical management (Figure 1).

**Figure 2:**
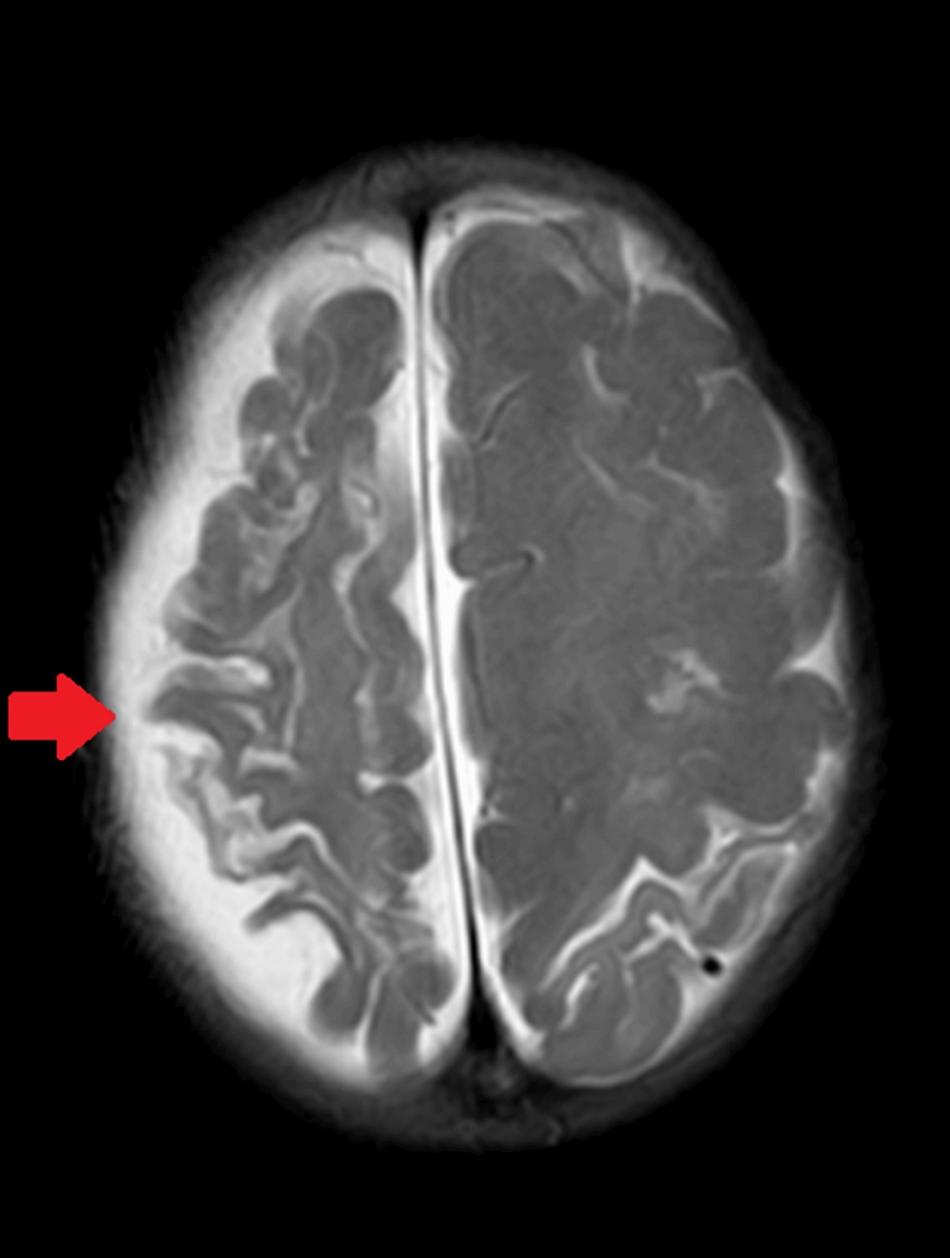
Left parietal-temporal infarct with cerebral palsy

Upon admission to the pediatric department, the medical team thoroughly assessed the child, re-evaluating the history and considering other findings. A CT scan was suggested, revealing a left parietal-temporal infarct with cerebral palsy. Consequently, an MRI scan was recommended for a more precise diagnosis (Figure 2).

**Figure 3:**
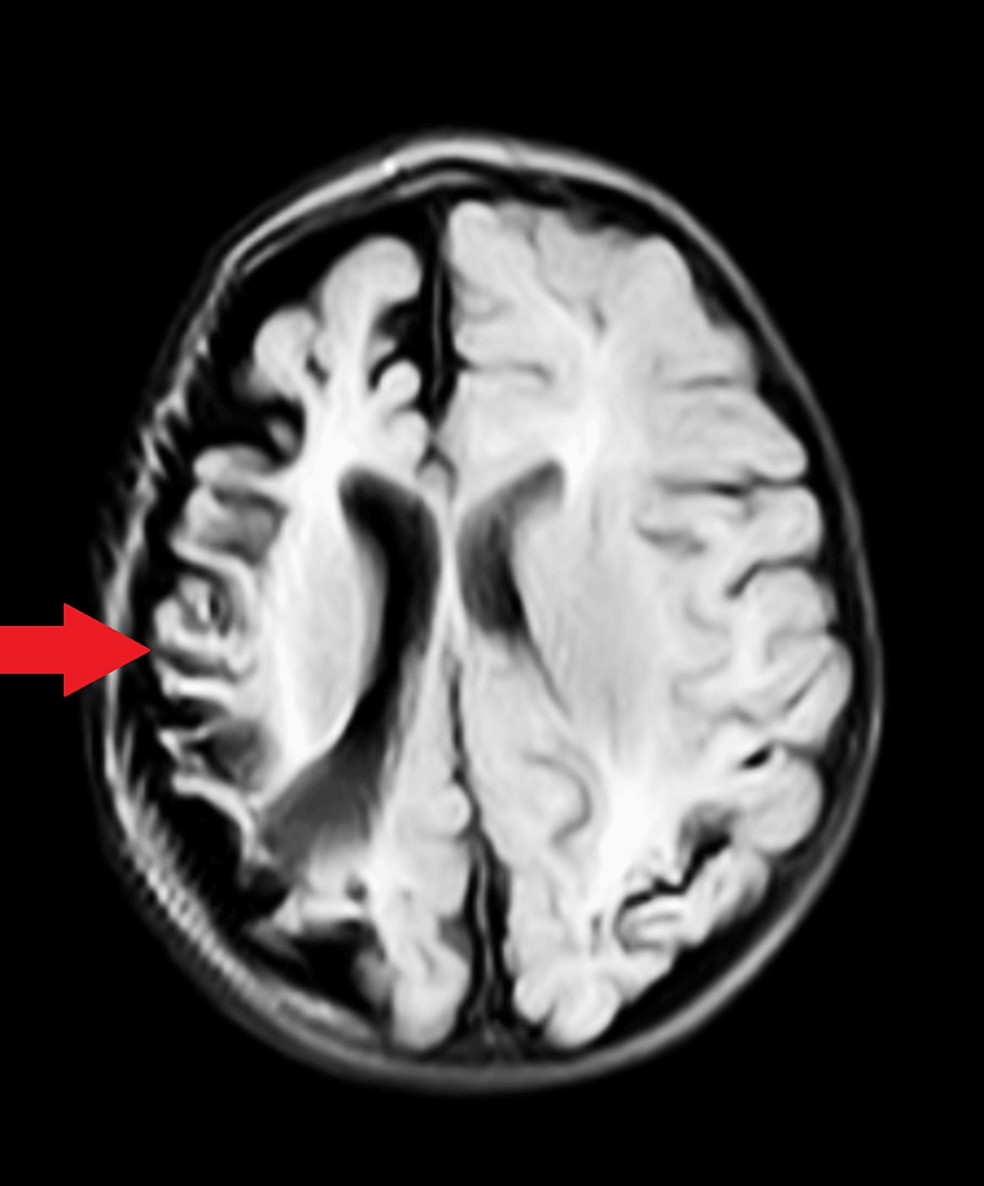
Atrophy of the right-sided cerebral hemisphere, ventricular enlargement, calvarial thickening on the ipsilateral side, and hyperpneumatization of the right frontal sinus

The MRI results indicated atrophy of the right-sided cerebral hemisphere, ventricular enlargement, calvarial thickening on the ipsilateral side, and hyperpneumatization of the right frontal sinus (Figure 3). The pediatrician diagnosed the child with DDMS. 

The child remained vitally stable with proper control of seizure crises and an adequate level of antiepileptic medication. Consequently, the medical team planned for discharge by continuing oral antiepileptic medication and advised follow-up care.

## Discussion

The presented case of DDMS in a 10-month-old child highlights the rarity of this condition in early infancy and underscores the challenges associated with its diagnosis and management. DDMS is a neurodevelopmental disorder characterized by cerebral hemiatrophy, ventricular enlargement, and calvarial thickening, usually identified in older children and adolescents [7]. However, this case contributes to the limited literature documenting the occurrence of DDMS in infants, emphasizing the importance of considering rare neurological disorders in the differential diagnosis of pediatric patients presenting with atypical symptoms. The etiology of DDMS remains multifactorial, with vascular insult, infection, and trauma implicated as potential causative factors [8]. The early onset of DDMS, in this case, following episodes of fever, raises questions about the role of infection or an inflammatory response in the pathogenesis of the syndrome. Further investigation into the specific triggers for DDMS in infancy is warranted to enhance our understanding of the underlying mechanisms.

Imaging plays a crucial role in confirming the diagnosis of DDMS; in this case, both CT and MRI scans provided valuable insights. The CT scan initially revealed a left parietal-temporal infarct with cerebral palsy, prompting further evaluation with MRI. The MRI results demonstrated atrophy of the right cerebral hemisphere, ventricular enlargement, calvarial thickening, and hyperpneumatization of the right frontal sinus, all consistent with the characteristic features of DDMS [9]. These findings underscore the significance of advanced imaging techniques in elucidating the structural abnormalities associated with DDMS and differentiating it from other neurological conditions. The management of DDMS involves a multidisciplinary approach, focusing on the underlying cause and symptom control. In this case, antiepileptic medications were successfully employed to control generalized tonic-clonic seizures, leading to the stabilization of the child's vitals. However, due to the rarity of DDMS in infancy, evidence-based guidelines for optimal management still need to be developed, necessitating individualized treatment plans [10].

The long-term prognosis for infants diagnosed with DDMS remains uncertain, and the development of neurological deficits may impact the child's overall quality of life. Regular follow-up care is essential to monitor the child's neurodevelopmental progress and adjust management strategies as needed [11]. The case presented here contributes to the existing knowledge base. It emphasizes the need for ongoing research to establish standardized diagnostic criteria and management protocols for DDMS in the pediatric population.

## Conclusions

In conclusion, the presented case of DDMS in a 10-month-old child underscores the rarity of this neurological disorder in early infancy. The diagnostic journey involved a comprehensive evaluation, incorporating clinical examination and advanced imaging modalities such as CT and MRI scans. The case raises intriguing questions about potential triggers and etiological factors, particularly given the onset of DDMS following episodes of fever. Successful seizure management with antiepileptic medication and the stabilization of vital signs highlight the importance of early recognition and intervention in pediatric DDMS cases. However, the absence of standardized guidelines for managing DDMS in infants emphasizes the need for individualized treatment plans and ongoing followup to monitor neurodevelopmental progress. While contributing to the limited literature on DDMS in infancy, this case report also underscores the challenges associated with diagnosis and management. Continued research is essential to expand our understanding of DDMS, establish standardized diagnostic criteria, and refine evidence-based management approaches, especially in the context of early infancy. Heightened clinical awareness, advanced imaging, and a multidisciplinary approach remain pivotal in navigating the complexities of this rare neurological disorder in pediatric patients.
